# Increased Risk of Temporomandibular Joint Disorder in Osteoporosis Patients: A Longitudinal Study

**DOI:** 10.3389/fendo.2022.835923

**Published:** 2022-03-31

**Authors:** So Young Kim, Dae Myoung Yoo, Mi Jung Kwon, Ji Hee Kim, Joo-Hee Kim, Soo-Hwan Byun, Bumjung Park, Hyo-Jeong Lee, Hyo Geun Choi

**Affiliations:** ^1^ Department of Otorhinolaryngology-Head and Neck Surgery, CHA Bundang Medical Center, CHA University, Seongnam, South Korea; ^2^ Hallym Data Science Laboratory, Hallym University College of Medicine, Anyang, South Korea; ^3^ Department of Pathology, Hallym University College of Medicine, Anyang, South Korea; ^4^ Department of Neurosurgery, Hallym University College of Medicine, Anyang, South Korea; ^5^ Division of Pulmonary, Allergy and Critical Care Medicine, Department of Medicine, Hallym University College of Medicine, Anyang, South Korea; ^6^ Department of Oral and Maxillofacial Surgery, Dentistry, Hallym University College of Medicine, Anyang, South Korea; ^7^ Research Center of Clinical Dentistry, Hallym University Clinical Dentistry Graduate School, Chuncheon, South Korea; ^8^ Department of Otorhinolaryngology-Head and Neck Surgery, Hallym University College of Medicine, Anyang, South Korea

**Keywords:** osteoporosis, temporomandibular joint disorders, risk factors, cohort studies, epidemiology

## Abstract

**Background:**

The objective of this study was to investigate the risk of temporomandibular joint disorder (TMD) in patients with osteoporosis.

**Methods:**

Data from the Korean National Health Insurance Service-Health Screening Cohort from 2002 to 2015 were collected. Participants ≥ 40 years old were enrolled, and the history of osteoporosis was evaluated. The 62,328 osteoporosis patients were matched for age, sex, income, and region of residence with 62,328 control participants. The occurrence of TMD was assessed in both the osteoporosis and control groups during the follow-up period. Stratified Cox proportional hazard analyses for TMD were conducted for the osteoporosis and control groups. The hazard ratios (HRs) of osteoporosis for TMD were further analyzed by age and sex subgroups.

**Results:**

A total of 1.2% (725/61,320) of the osteoporosis patients and 0.6% (339/61,320) of the control participants had TMD (P<0.001). Osteoporosis was associated with an elevated HR of TMD (adjusted HR=1.96, 95% CI=1.72–2.23, P<0.001). Among the age and sex subgroups, the < 60-year-old mal\e group demonstrated an adjusted HR of osteoporosis for TMD as high as 4.47 (95% CI=1.17–17.12, P=0.029). Other age and sex subgroups also showed a higher HR for TMD associated with osteoporosis (adjusted HR=2.30, 95% CI=1.90–2.78, P<0.001 for the ≥ 60-year-old female group).

**Conclusion:**

Osteoporosis was related to a higher risk of TMD in the adult population. A prominent association of osteoporosis with TMD was noted in middle-aged men and older women.

## Introduction

Temporomandibular disorder (TMD) is a spectrum of diseases characterized by pain and limited function of the temporomandibular joint (TMJ) and the masticatory apparatus (1). TMD includes diseases related to the TMJ, such as degenerative diseases and disc displacements, and pain-related diseases such as myalgia, headache, and arthralgia (2). Thus, TMD assessments include the diagnosis of pain-related disorders and intra-articular disorders of the TMJ (3). TMD predominantly affects women and the middle-aged population (4). Numerous comorbidities, including degenerative arthritis, headache, depression, and autoimmune disorders, have been positively associated with TMD (4).

Because TMD is associated with degenerative changes in the TMJ, particularly the bony components, in approximately 22.0% of patients (5, 6), several previous studies have suggested that an increased risk of degenerative changes in the TMJ is associated with decreased bone mineral density (BMD) (7, 8). Radiographic changes in the TMJ, such as erosion, flattening, and osteophytes, are correlated with low levels of a bone formation marker, Type I collagen telopeptide fragments (r=[-0.217], n=95, P=0.041) (8). Dervis E suggested that osteoporosis-related bony resorption occurs in the condylar and temporal components of the TMJ (7). However, degenerative changes in the TMJ are not correlated with symptomatic TMD, which is accompanied by pain and limitations in the motion of the TMJ (5). Thus, it is presumed that factors in addition to bone loss in the TMJ may contribute to the link between osteoporosis and TMD.

Osteoporosis is a skeletal disease characterized by compromised bone strength and increased susceptibility to fracture (9). These changes can be attributed to defects in the microarchitecture or the intrinsic properties of bone tissue, which are influenced by metabolic and endocrine changes (10). A number of risk factors, including immobility, menopause, smoking, and type 2 diabetes, have been reported to be related to the risk of osteoporosis (11, 12).

We hypothesized that osteoporosis could be associated with an increased risk of TMD. To test this hypothesis, the occurrence of TMD in osteoporosis patients was compared with that in matched control participants. Although several previous studies investigated the relationship of osteoporosis with TMD, their samples were limited to elderly or female populations (13–15). In addition, comorbidities and lifestyle factors could influence the association of osteoporosis with TMD and should be considered potential confounders. The present study improved upon previous studies by using a large cohort population and adjusting for the aforementioned potential confounders.

## Methods

### Study Population

The ethics committee of Hallym University (2019-10-023) approved the present research. The Institutional Review Board provided an exemption for the requirement of written informed consent. The Korean National Health Insurance Service-Health Screening Cohort (NHIS-HEALS) were analyzed. The NHIS-HEALS is composed of all insured Koreans with ≥ 40 years old who underwent biannual health examinations without cost. Because all Koreans are legally registered in NHIS with respective registrations number, there was little concern on the possible missing or overlapping population. A detailed description of the NHIS-HEALS data are provided elsewhere (**S1** description) (16).

### Definition of Osteoporosis (Independent Variable)

Osteoporosis was defined as in previous studies (17, 18). In brief, ≥ 2 instances of diagnostic codes M80 (osteoporosis with pathological fracture), M81 (osteoporosis without pathological fracture), and M82 (osteoporosis in diseases classified elsewhere) from 2002 through 2015 and bone densitometry using dual energy X-ray absorptiometry (DEXA) or a DEXA CT scan (Claim code: E7001-E7004) (17, 18).

### Definition of Temporomandibular Joint Disorder (Dependent Variable)

TMD was defined as a diagnosis of K07.6 (temporomandibular joint disorders). Participants who visited the clinic ≥ 2 times and were diagnosed with code K07.6 were included (19). The date of outcome event (diagnosis of TMD) was defined as the date of initial diagnosis of TMD.

### Participant Selection

**Figure 1:**
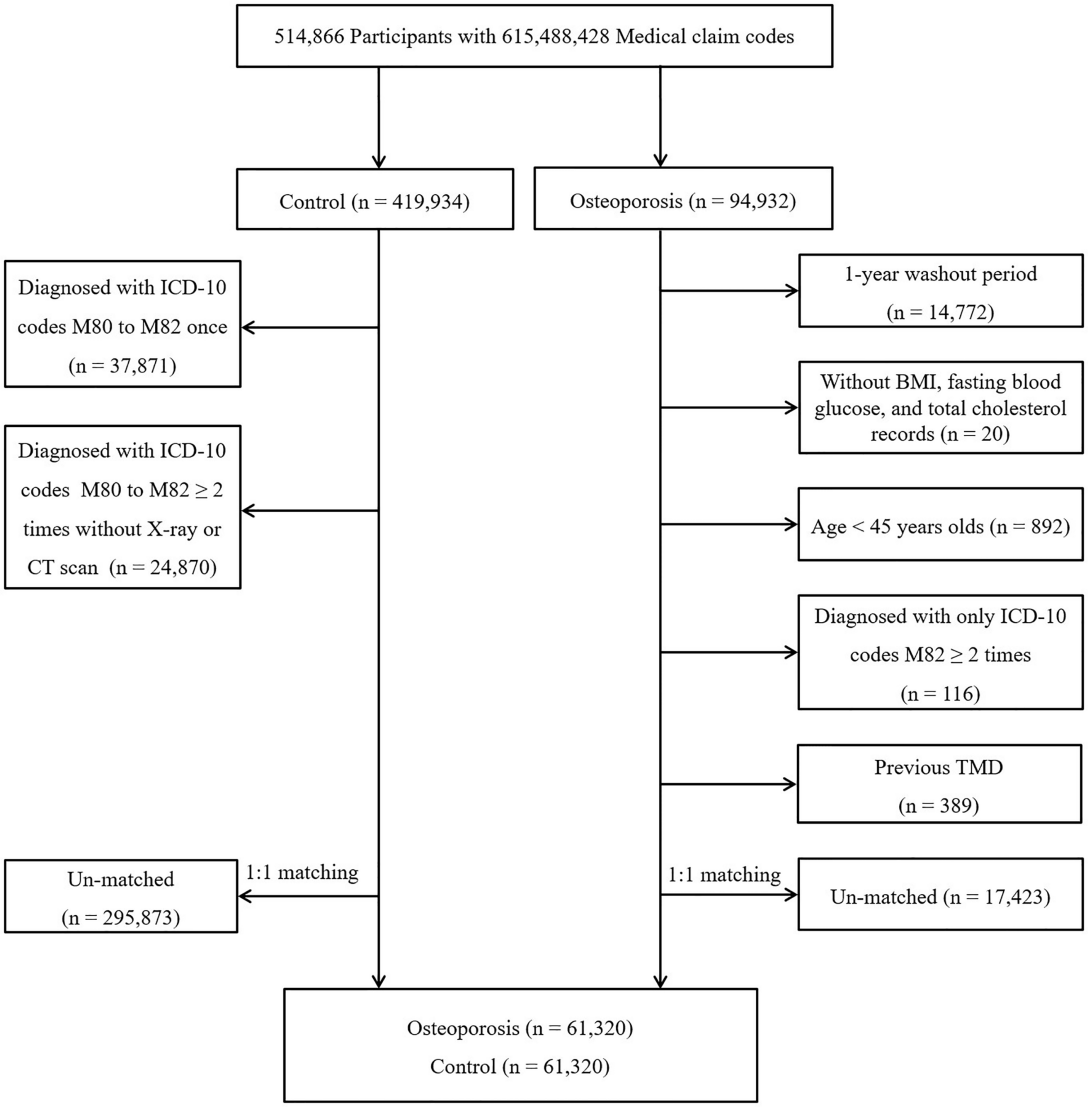
A schematic illustration of the participant selection process used in the present study. From a total of 514,866 participants, 61,320 osteoporosis participants were 1:1 matched with 61,320 control participants for age, sex, income, and region of residence.

Osteoporosis participants were enrolled from a group of 514,866 participants with 615,488,428 medical claim codes from 2002 through 2015 (n=94,932). Participants were enrolled in the control group if they were not defined as having osteoporosis from 2002 through 2015 (n=419,934). To select participants who were diagnosed with osteoporosis for the first time, those who were diagnosed in 2002 were excluded (washout period, n=14,772). Participants with osteoporosis who did not have body mass index (BMI), fasting blood glucose, or total cholesterol data were excluded (n=20). Participants with osteoporosis who were under 45 years old (n = 892) and who were diagnosed with only ICD-10 code M82 ≥ 2 times (n = 116) were excluded. Control participants were excluded if they were diagnosed with codes M80, M81, or M82 once (n=37,871). Control participants who were diagnosed with codes M80, M81, and M82 ≥ 2 times without DEXA or DEXA CT scans (claim code: E7001-E7004) were excluded (n=24,870). Age, sex, income, and region of residence were matched between the participants in the osteoporosis and control groups. The control participants were randomly selected. To prevent selection bias when selecting the matched participants, the control participants were sorted using a random number order and were then selected from top to bottom. The index date was defined as the date of diagnosis with osteoporosis. The control participants had the same index date as their matched participants with osteoporosis. Thus, the date of the osteoporosis patient’s osteoporosis diagnosis was set as the index date of his or her matched control participant. Participants who died before the index date were excluded. Participants who had a history of TMD before the index date were excluded (n=389 in osteoporosis group). During the matching procedure, 17,423 osteoporosis patients and 295,873 control participants were excluded. Finally, 61,320 osteoporosis patients were 1:1 matched with 61,320 control participants (**Figure 1**).

### Covariates

Age groups were classified at 5-year intervals. Income level was classified according to the Korea National Health Insurance premium and ranged from 2 (the lowest 20%) to 5 (the highest 20%) for the entire Korean population with health insurance. The participants who were medical aid beneficiaries were classified as the lowest income level (class 1) (20, 21). The region of residence was divided into urban and rural areas (17).

Smoking status and the frequency of alcohol consumption were classified as categorical variables. Smoking status was divided into nonsmokers, past smokers, and current smokers based on a questionnaire. Participants with a history of smoking more than 100 cigarettes but did not currently smoke were classified as past smokers. BMI (kg/m^2^), systolic blood pressure, diastolic blood pressure, fasting blood glucose, and total cholesterol were categorized using the normal range as a reference. Medical history was considered using the Charlson Comorbidity Index (CCI) (17).

### Statistical Analyses

Chi-square tests were used to compare the general characteristics of the osteoporosis and control groups.

Stratified Cox proportional hazard models were used to assess the hazard ratios (HRs) and 95% confidence intervals (CIs) for TMD in the osteoporosis group. Crude and adjusted analyses (for obesity, smoking, alcohol consumption, systolic blood pressure, diastolic blood pressure, fasting blood glucose, total cholesterol and CCI scores) were conducted. Stratification was conducted for age, sex, income, and region of residence. Kaplan–Meier analysis and the log-rank test were used to estimate the cumulative probability of TMD in the osteoporosis group. The zph test was conducted to test the proportional hazards assumption for a Cox regression model. The P-value was 0.4528. Subanalyses were conducted by age and sex.

The E-value was evaluated to assume the minimum strength of association that an unmeasured confounder would need to have with both the treatment and outcome to fully explain away a specific treatment-outcome association, conditional on the measured covariates (22, 23).

All analyses were two tailed. P < 0.05 was defined as statistically significant. SAS version 9.4 (SAS Institute, Inc., Cary, NC, USA) was used.

## Results

The mean follow-up period was 99.95 (standard deviation [SD] = 41.82) months for osteoporosis patients and 96.35 (SD = 43.52) months for the control participants. During the follow-up period, 7.73% of the osteoporosis patients and 12.01% of the control participants died.

A total of 1.2% (715/61,320) and 0.6% (339/61,320) of the osteoporosis patients and the control participants, respectively, had TMD (P<0.001, **Table 1**). The osteoporosis group showed higher rates of underweight and lower rates of obesity I and II than the control group (P<0.001). Current smoking, frequent alcohol consumption, abnormal systolic and diastolic blood pressure, abnormal fasting blood glucose, and abnormal total cholesterol were less frequent in the osteoporosis group than in the control group (all P<0.001). The distribution of CCI scores differed between the osteoporosis group and the control group (P<0.001). Age and sex did not show significant interaction with TMD (P = 0.053 for age and P = 0.580 for sex, **Table S2**).

**Table 1:** General Characteristics of Participants.

Characteristics	Total participants		
	Osteoporosis (n, %)	Control (n, %)	P-value
Age (years old)			1.000
45-49	4,400 (7.2)	4,400 (7.2)	
50-54	9,938 (16.2)	9,938 (16.2)	
55-59	11,485 (18.7)	11,485 (18.7)	
60-64	13,452 (21.9)	13,452 (21.9)	
65-69	9,517 (15.5)	9,517 (15.5)	
70-74	6,625 (10.8)	6,625 (10.8)	
75-79	4,057 (6.6)	4,057 (6.6)	
80-84	1,584 (2.6)	1,584 (2.6)	
85+	262 (0.4)	262 (0.4)	
Sex			1.000
Male	9,279 (11.0)	9,279 (11.0)	
Female	52,041 (84.9)	52,041 (84.9)	
Income			1.000
1 (lowest)	11,571 (18.9)	11,571 (18.9)	
2	9,299 (15.2)	9,299 (15.2)	
3	9,944 (16.2)	9,944 (16.2)	
4	12,541 (20.4)	12,541 (20.4)	
5(highest)	17,965 (29.3)	17,965 (29.3)	
Region of residence			
Urban	25,292 (41.2)	25,292 (41.2)	
Rural	36,028 (58.7)	36,028 (58.7)	
Obesity^†^			<0.001*
Underweight	2,037 (3.3)	1,477 (2.4)	
Normal	23,575 (38.4)	20,386 (33.5)	
Overweight	16,096 (26.2)	16,076 (26.2)	
Obese I	17,874 (29.1)	20,662 (33.7)	
Obese II	1,738 (2.8)	2,719 (4.4)	
Smoking status			<0.001*
Nonsmoker	55,499 (90.5)	54,969 (89.7)	
Past smoker	2,392 (3.9)	2,421 (3.9)	
Current smoker	3,429 (5.6)	3,930 (6.4)	
Alcohol consumption			<0.001*
<1 time a week	53,490 (87.2)	52,853 (86.2)	
≥1 time a week	7,830 (12.8)	8,467 (13.8)	
Systolic blood pressure			<0.001*
<120 mmHg	19,369 (31.6)	17,179 (28.0)	
120-139 mmHg	27,364 (44.6)	26,800 (43.7)	
≥140 mmHg	14,587 (23.8)	17,341 (28.3)	
Diastolic blood pressure			<0.001*
<80 mmHg	29,369 (47.9)	26,875 (43.8)	
80-89 mmHg	20,525 (33.4)	20,961 (34.2)	
≥90 mmHg	11,426 (18.6)	13,484 (22.0)	
Fasting blood glucose			<0.001*
<100 mg/dL	42,628 (69.5)	39,649 (64.7)	
100-125 mg/dL	14,650 (23.9)	15,677 (25.6)	
≥126 mg/dL	4,042 (6.6)	5,994 (9.8)	
Total cholesterol			<0.001*
<200 mg/dL	29,381 (47.9)	28,671 (46.8)	
200-239 mg/dL	21,707 (35.4)	21,882 (35.7)	
≥240 mg/dL	10,232 (16.7)	10,767 (17.6)	
CCI score			<0.001*
0	38,137 (62.2)	40,579 (66.1)	
1	10,317 (16.8)	8,642 (14.1)	
2	5,888 (9.6)	5,063 (8.3)	
3	3,077 (5.0)	2,792 (4.5)	
≥ 4	3,901 (6.4)	4,244 (6.9)	
Temporomandibular joint disorder	715 (1.2)	339 (0.6)	<0.001*

CCI, Charlson comorbidity index.

^*^Chi-square test. Significance at P < 0.05.

^†^Obesity (BMI, body mass index, kg/m^2^) was categorized as < 18.5 (underweight), ≥ 18.5 to < 23 (normal), ≥ 23 to < 25 (overweight), ≥ 25 to < 30 (obese I), and ≥ 30 (obese II).

**Figure 2:**
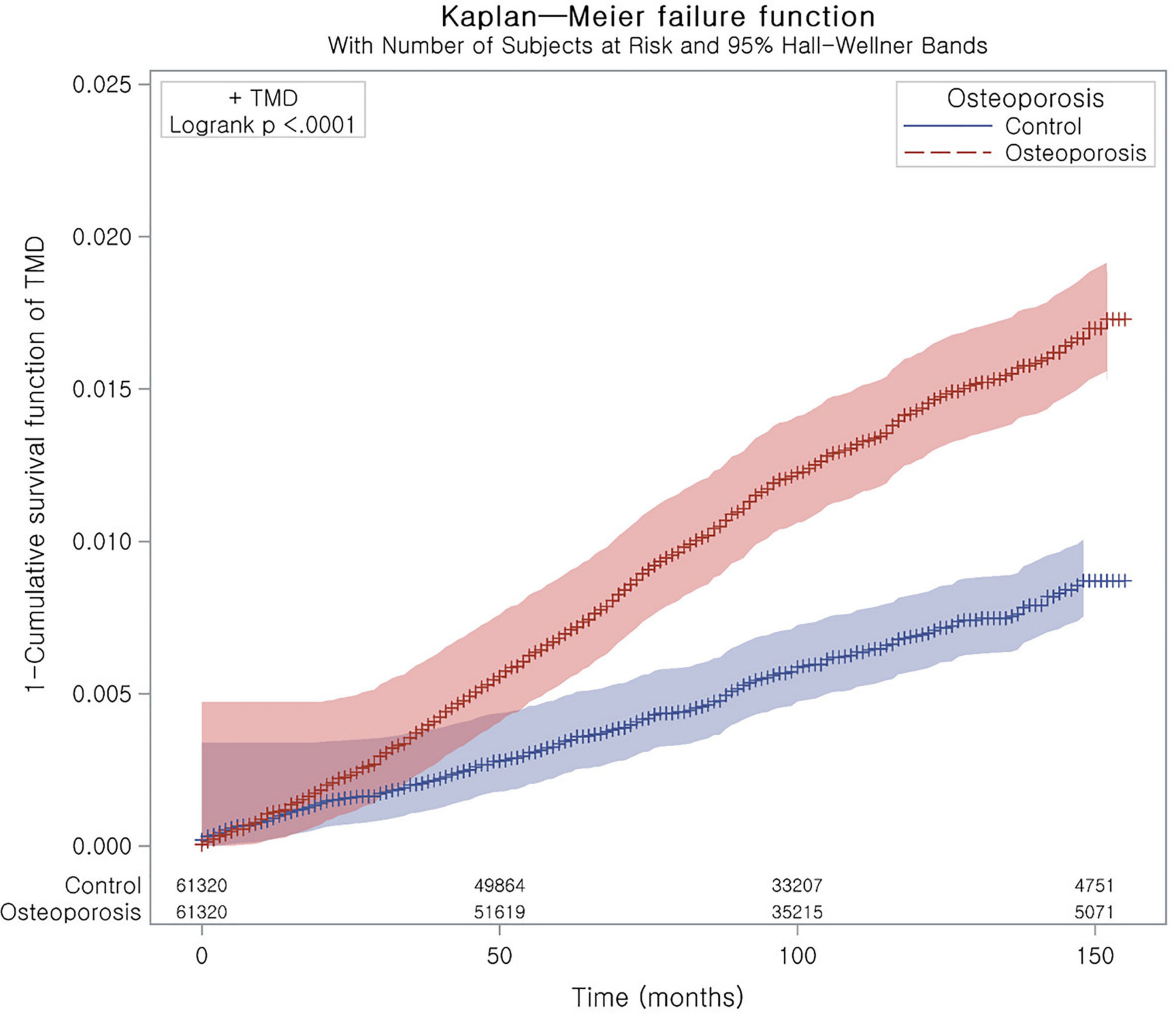
Kaplan–Meier curve of TMD with osteoporosis. It represents a curve of 1 – survival function.

Osteoporosis was associated with an HR of 1.96 for TMD in the adjusted model (95% CI=1.72–2.23, P<0.001, **Table 2** and **Figure 2**). The E-value of TMD for osteoporosis was estimated 3.33 (CI = 2.83). The E-value was higher than previously reported odds of non-specific symptoms for TMD (adjusted OR = 2.46, CI = 1.25) (24). However, it was lower than previously reported odds of malocclusion for TMD (adjusted OR = 8.3 – 12.4) (25). All age and sex subgroups demonstrated higher HRs for TMD with osteoporosis. The adjusted HR for TMD with osteoporosis was highest in the < 60-year-old male group (adjusted HR=4.47, 95% CI=1.17–17.12, P=0.029). The ≥ 60-year-old female group showed the second highest HR for TMD with osteoporosis (adjusted HR=2.30, 95% CI=1.90–2.78, P<0.001). The ≥ 60-year-old male groups had HR for TMD with osteoporosis of 1.66 (95% CI=1.12–2.45).

**Table 2:** Crude and adjusted hazard ratios (95% confidence interval) for temporomandibular joint disorder in osteoporosis and control groups according to age and sex.

Independent variables	TMD/participants (n, %)	Follow-up duration(Person-Years)	IR per 1000 Person-Years	IRD per 1000 person-years(95% CI)	Hazard ratios (95% CI)				E-value
					Crude^†^	P-value	Adjusted^†‡^	P-value	Estimate (CI)
Total participants (n = 122,640)									
Osteoporosis	715/30,660 (1.2)	482,239	1.48	0.75 (0.62 to 0.89)	2.03 (1.79-2.32)	<0.001*	1.96 (1.72-2.23)	<0.001*	3.33 (2.83)
Control	339/30,660 (0.6)	464,075	0.73		1.00		1.00		
Age < 60 years old, men (n = 2,646)									
Osteoporosis	10/1,323 (0.8)	9,022	1.11	0.78 (-0.01 to 1.56)	3.40 (0.93-12.35)	0.063	4.47 (1.17-17.12)	0.029*	8.41 (1.62)
Control	3/1,323 (0.2)	9,010	0.33		1.00		1.00		
Age < 60 years old, women (n = 49,000)									
Osteoporosis	252/24,500 (1.0)	200,236	1.26	0.52 (0.33 to 0.72)	1.71 (1.40-2.10)	<0.001*	1.65 (1.35-2.03)	<0.001*	2.69 (2.04)
Control	146/24,500 (0.6)	198,664	0.73		1.00		1.00		
Age ≥ 60 years old, men (n = 15,912)									
Osteoporosis	72/7,956 (0.9)	41,187	1.75	0.74 (0.22 to 1.25)	1.74 (1.18-2.56)	0.005*	1.66 (1.12-2.45)	0.011*	2.71 (1.49)
Control	40/7,956 (0.5)	39,547	1.01		1.00		1.00		
Age ≥ 60 years old, women (n = 55,082)									
Osteoporosis	381/27,541 (1.4)	231,794	1.64	0.95 (0.75 to 1.15)	2.40 (1.98-2.89)	<0.001*	2.30 (1.90-2.78)	<0.001*	4.03 (3.21)
Control	150/27,541 (0.5)	216,854	0.69		1.00		1.00		

CCI, Charlson Comorbidity Index.

^*^Stratified Cox proportional hazard regression model, Significance at P < 0.05.

^†^Models were stratified by age, sex, income, and region of residence.

^‡^The model was adjusted for obesity, smoking, alcohol consumption, systolic blood pressure, diastolic blood pressure, fasting blood glucose, total cholesterol and CCI scores.

## Discussion

Osteoporosis was associated with a higher HR for TMD in the adult population. The increased risk of TMD associated with osteoporosis was highest in the middle-aged male population, followed by the elderly female population.

A few previous studies evaluated the relationship between osteoporosis and the presence of TMD (15, 26, 27). Osteoporosis was related to a 2.56-fold higher risk of TMD in patients with chronic obstructive pulmonary disease (95% CI=1.38–4.78, P<0.001) (26). Another retrospective study reported a 9.00-fold higher risk of TMD associated with osteoporosis in patients with chronic obstructive pulmonary disease and vertebral compression fracture (95% CI=3.01–48.60, P<0.001) (27). On the other hand, a cross-sectional study demonstrated no significant association between the clinical diagnosis of TMD and osteoporosis or osteopenia in elderly individuals (15). However, the study population was relatively small (n= 44), and the age range was limited to 80 years or older (15). The present results improved on previous studies by including middle-aged as well as elderly age groups and both sexes.

The compromised TMJ architecture and poor bone quality in osteoporosis could mediate the presence of TMD in osteoporosis patients. Although the assertion is controversial, the reduced bone quality in osteoporosis patients could be associated with TMJ degeneration (7, 8, 28). Radiographic changes in the TMJ, such as fewer occluding pairs of teeth and the use of prostheses, showed inverse relationships with BMD and the bone turnover markers type 1 collagen telopeptide fragments, C-telopeptide crosslaps of type 1 collagen, and 25 (OH)D (8). In addition, a cone-beam CT study revealed a higher prevalence of osteoporosis or osteopenia in patients with TMJ changes, including flattening, erosion, sclerosis, subchondral cysts, and osteophytes (28). However, there is some controversy regarding the correlation between skeletal BMD and mandibular BMD (29). In addition, there have been conflicting results regarding the association between TMJ changes and TMD (15). A cross-sectional study reported that there was no relation of osteoporosis or osteopenia with either radiographic alteration of the condyle or orofacial pain (15). Thus, the impact of osteoporosis on TMD might not be solely dependent on TMJ degenerative changes.

Hormonal imbalance, such as disturbances in the hypothalamic–pituitary–adrenal (HPA) axis and estrogen, may induce TMD in osteoporosis patients. Osteoporosis can accompany changes in the levels of several hormones, including increased cortisol and decreased estrogen (30). Hormonal imbalance could cause TMD *via* neuroendocrine dysregulation of pain sensation and TMJ changes. TMD patients demonstrate an upregulated hypothalamic–pituitary–adrenal (HPA) axis with high cortisol compared with control participants (31, 32). Similar to the situations of other stress-related and chronic pain disorders, the presence of an upregulated HPA axis has been suggested in TMD patients (33). In addition, psychological stress can induce facial pain sensation and can contribute to TMD (33, 34). Impaired negative feedback of the HPA axis has been suggested in TMD and in major depression (31). In summary, both psychological and neuroendocrine impairments related to osteoporosis may be implicated in TMD (33).

The low estrogen level of postmenopausal women with osteoporosis could trigger TMD. Osteoporosis in postmenopausal women is primarily associated with the loss of the protective effects of estrogen in the context of osteoporosis. Low estrogen levels can impact both TMJ components and pain modulation (35, 36). The fluctuation of estrogen levels during the reproductive period has been reported to aggravate facial pain, and the decline in estrogen levels during menopause could increase degenerative changes in the TMJ (36). In addition, estrogen has been thought to regulate TMJ inflammation and nociceptive responses *via* peripheral and central nervous pathways (37, 38). Therefore, osteoporosis in older women may be linked with low estrogen levels, and the low estrogen levels in these patients can result in TMD through the degeneration of the TMJ and increased pain responses. In the present study, older women showed a more than twofold higher risk of TMD associated with osteoporosis.

On the other hand, the association of osteoporosis with TMD in the older male group was not as high as that in the older female group in the current study. In addition to the minimal effects of estrogen in older men, the symptoms of TMD were reported to be mild and self-limiting in the older population (5). Thus, there might be fewer TMD patients who require medical treatment in the older male group. However, in the middle-aged male group, the association between osteoporosis and TMD was higher than in the other age and sex subgroups in this study. This can be explained by sex differences in the pathophysiology and causes of osteoporosis (39). Although total bone mass is generally higher in males than females, trabecular bone loss begins earlier, and the cortical thinning rate is higher in males than females (40, 41). In addition, osteoporosis secondary to extraskeletal diseases accounts for approximately 65% of cases in males, compared to approximately 20-40% in females (42, 43). The higher fragility of trabecular bone and possible underlying comorbid conditions could impose a higher risk of TMD related to osteoporosis in the middle-aged male population in this study.

This study used data from a large nationwide population and included a control group of participants who were matched for demographic and socioeconomic factors with osteoporosis patients. In addition, lifestyle factors (obesity, smoking, and alcohol consumption), laboratory measures (blood pressure, fasting blood glucose, and total cholesterol) and comorbidities (described using the CCI score) were considered as covariates to attenuate possible confounding effects. Considering the age and sex differences in both osteoporosis and TMD, age and sex subgroups were analyzed separately, and the results revealed a stronger association of osteoporosis with TMD in middle-aged men and older women. However, this study had some limitations related to the accuracy of the diagnosis of osteoporosis and TMD because the BMD and radiologic findings for TMJ are not accessible in the Korean National Health Insurance Service-Health Screening Cohort data. Thus, the severity and types of osteoporosis and TMD were heterogeneous among patients. The site of osteoporotic fractures (M80) is not specified in the Korean National Health Insurance Service-Health Screening Cohort data. The tools used to diagnose osteoporosis were not unified and included both DEXA and DEXA CT scans. In addition, medication histories related to osteoporosis and TMD were not considered. For instance, patients with Cushing syndrome due to excess cortisol could have an increased risk of both osteoporosis and TMD. For lifestyle factors, such as smoking and alcohol consumption, although the cohort data was collected by Korean government, there has been no study evaluated the validity of these variables. Finally, although numerous covariates were adjusted, there could be some potential additional confounders, such as stress level. The E-value analyses indicated as high as 3.33 of E-value of TMD for osteoporosis, which was higher than previously reported odds of nonspecific symptoms for TMD, while lower than the odds of malocclusion for TMD (24, 25).

## Conclusions

Osteoporosis was associated with an increased risk of TMD in adults. Middle-aged male and older female populations showed a strong relationship between osteoporosis and TMD.

## Data Availability Statement

The datasets presented in this article are not readily available because releasing of the data by the researcher is not legally permitted. All data are available from the database of the Korea Center for Disease Control and Prevention. The Korea Center for Disease Control and Prevention allows data access, at a particular cost, for any researcher who promises to follow the research ethics. The data of this article can be downloaded from the website after agreeing to follow the research ethics. Requests to access the datasets should be directed to Korea Center for Disease Control and Prevention.

## Ethics Statement

The ethics committee of Hallym University (2019-10-023) permitted the present research. The Institutional Review Board provided an exemption for the requirement of written informed consent. The ethics committee waived the requirement of written informed consent for participation.

## Author Contributions

HC designed the study. DY, MK, and HC analyzed the data. SK, JK, J-HK, S-HB, and HC drafted and revised the paper. HC drew the figures. All authors read and agreed to the published version of the manuscript.

## Funding

This work was supported in part by research grants (NRF-2018-R1D1A1A02085328 and 2021-R1C1C1004986) from the National Research Foundation (NRF) of Korea. The funders had no role in the design of the study; the collection, analysis, and interpretation of the data; or the writing of the manuscript.

## Conflict of Interest

The authors declare that the research was conducted in the absence of any commercial or financial relationships that could be construed as a potential conflict of interest.

## Publisher’s Note

All claims expressed in this article are solely those of the authors and do not necessarily represent those of their affiliated organizations, or those of the publisher, the editors and the reviewers. Any product that may be evaluated in this article, or claim that may be made by its manufacturer, is not guaranteed or endorsed by the publisher.
