# Supplementary material for: Increased Risk of Temporomandibular Joint Disorder in Osteoporosis Patients: A Longitudinal Study
Source: Front Endocrinol (Lausanne). 2022 Mar 31;13:835923. doi: 10.3389/fendo.2022.835923 (PMC9008302; doi:10.3389/fendo.2022.835923)
Supplement: Supplementary file 1 [file DataSheet_1.docx]

## **S1 description: Study Population and Data Collection**

This national cohort study relied on data from the Korean National Health Insurance Service-Health Screening Cohort (NHIS-HEALS) (1). The Korean National Health Insurance Service (NHIS) randomly selects approximately 10% of individuals who underwent health examinations from 2002 to 2003 (n = ~515,000) directly from the entire population (n = ~5,150,000). Age and sex specific distributions of the cohort population are described online (2). The details of the methods used to perform these procedures are provided by the National Health Insurance Sharing Service (3).

All insured Koreans who are at least 40 years old and their dependents undergo no-cost biannual health examinations (4). Each examinee must complete a standard questionnaire in for this health screening program (4). Because all Korean citizens are recognized by a 13-digit resident registration number from birth to death, exact population statistics can be determined using this database. It is mandatory for all Koreans to enroll in the NHIS. All Korean hospitals and clinics use the 13-digit resident registration number to register individual patients in the medical insurance system. Therefore, the risk of overlapping medical records is minimal, even if a patient moves from one place to another. Moreover, all medical treatments in Korea can be tracked without exception using the Korean Health Insurance Review & Assessment (HIRA) system. In Korea, providing a notice of death to an administrative entity is legally required before a funeral can be held, and the cause and date of death are recorded by medical doctors on a death certificate.

This cohort database includes (i) personal information, (ii) health insurance claim codes (procedures and prescriptions), (iii) diagnostic codes using the International Classification of Disease-10 (ICD-10), (iv) death records from the Korean National Statistical Office (using the Korean Standard Classification of disease), (v) socioeconomic data (residence and income), (vi) medical examination data (vii) health examination data (body mass index [BMI], drinking and smoking habits, blood pressure, urinalysis, hemoglobin, fasting glucose, lipid parameters, creatinine, and liver enzymes) for each participant over the period from 2002 to 2013 (3, 4).

**References**

1. Lee J, Lee JS, Park SH, Shin SA, Kim K. Cohort Profile: The National Health Insurance Service-National Sample Cohort (NHIS-NSC), South Korea. Int J Epidemiol. 2017;46(2):e15.

2. Kaushal N, Keith N, Aguinaga S, Hagger MS. Social Cognition and Socioecological Predictors of Home-Based Physical Activity Intentions, Planning, and Habits during the COVID-19 Pandemic. Behav Sci (Basel). 2020;10(9).

3. <http://nhiss.nhis.or.kr/>.

4. Song SO, Jung CH, Song YD, Park CY, Kwon HS, Cha BS, et al. Background and data configuration process of a nationwide population-based study using the korean national health insurance system. Diabetes Metab J. 2014;38(5):395-403.
