# Supplementary material for: Increased Risk of Temporomandibular Joint Disorder in Osteoporosis Patients: A Longitudinal Study
Source: Front Endocrinol (Lausanne). 2022 Mar 31;13:835923. doi: 10.3389/fendo.2022.835923 (PMC9008302; doi:10.3389/fendo.2022.835923)
Supplement: Supplementary file 2 [file DataSheet_2.docx]

**Supplement Table S2** P-value of interaction according to Age and Sex

| Covariate | P-value |
| --- | --- |
| Age (< 60 vs ≥ 60) | 0.053 |
| Sex | 0.580 |
